# The role of stem cells in the prevention and treatment of radiation‐induced xerostomia in patients with head and neck cancer

**DOI:** 10.1002/cam4.609

**Published:** 2016-02-16

**Authors:** Daan Nevens, Sandra Nuyts

**Affiliations:** ^1^ Radiation OncologyLeuven Cancer InstituteUniversity Hospitals Leuven and Department of OncologyKU LeuvenHerestraat 493000LeuvenBelgium

**Keywords:** Parotid gland, radiotherapy, salivary gland hypofunction, salivary gland stem cell, xerostomia

## Abstract

Xerostomia is an important complication following radiotherapy (RT) for head and neck cancer. Current treatment approaches are insufficient and can only temporarily relieve symptoms. New insights into the physiopathology of radiation‐induced xerostomia might help us in this regard. This review discusses the current knowledge of salivary gland stem cells in radiation‐induced xerostomia and their value in the prevention and treatment of this complication. Salivary gland stem cell transplantation, bone marrow‐derived cell mobilization, molecular regulation of parotid stem cells, stem cell sparing RT, and adaptive RT are promising techniques that are discussed in this study.

## Introduction

Radiotherapy (RT) is an important treatment modality in the management of head and neck cancer (HNC). RT can be used as a single modality or in combination with surgery and/or chemotherapy (CT). One of the most frequently reported side effects following RT in patients with HNC is hyposalivation and subsequent xerostomia due to co‐irradiation of the salivary glands 1, 2. Xerostomia significantly impairs the patient's quality of life due to the many secondary effects such as impairment of taste, swallowing, and speech 2, 3, 4, 5, 6. Furthermore, the oral mucosa can become dry and atrophic, leading to frequent ulceration 6.

Approximately 40% of patients with HNC will experience xerostomia to some degree after treatment with RT 7, 8, 9, 10, 11. The introduction of new techniques such as parotid‐sparing intensity‐modulated RT (IMRT) has resulted in less xerostomia; however, toxicity outcome is still far from perfect 12. The Oncology and Radiotherapy Group for Head and Neck Cancer (GORTEC) proposed to evaluate prospectively acute and late toxicities, locoregional control, and overall survival for patients treated for head and neck cancer (HNC) with IMRT and bilateral neck irradiation in the GORTEC 2004–03 study. It was reported that 16.1% of patients treated with parotid‐sparing IMRT experienced severe xerostomia 18 months after the completion of treatment 13.

Xerostomia can occur early during RT treatment. Depending on the localization of the tumor and the radiation portals, a rapid decrease of the salivary flow rate is observed during the first week of RT, after which there is a continuing gradual decrease to less than 10% of the initial flow rate. This early reaction is unforeseen because the excretory, acinar cells in the salivary glands have a slow mitotic rate; a fast response to RT is therefore not expected 6, 14. It is suggested that early damage may be due to damage to the plasma membrane of acinar cells, compromising the receptor‐mediated signaling pathways of water excretion. No immediate cell death takes place. Late damage, on the other hand, may be explained by (DNA) damage to the salivary gland stem cells (SCs) and subsequent lack of proper cell renewal 15, 16.

A review of published studies suggested that severe xerostomia can be avoided if either the mean dose to both parotid glands is less than 25 Gy or one parotid gland is spared to a mean dose of less than 20 Gy 17. GORTEC 2004‐03 showed that a mean dose administered to the spared parotid below 28 Gy led to significantly less severe xerostomia 13.

Following RT, salivary gland recovery is dependent on the radiation dose and on the number of remaining viable SCs 6. Increasing the regenerative potential of salivary glands by SC therapy after irradiation should be able to restore tissue homeostasis 6. Gaining knowledge in the field of salivary gland SCs may thus provide means of preventing late xerostomia or could lead to new treatment strategies to improve regeneration of these cells after RT. These new treatment approaches are in demand because the current clinical management of xerostomia is often difficult and brings in many cases no substantial relief for the patient 18.

In 2006, our research group published a literature review on radiation‐induced xerostomia in patients with head and neck cancer 2. Since this review was published, there has been extensive research in the field of xerostomia prevention and treatment after RT in head and neck cancer. A substantial proportion of this research focused on the link between salivary gland SCs and xerostomia. Research on salivary gland SCs and treatment strategies regarding these cells is scattered in literature. Therefore, we present in this review an overview of new findings in this field, with emphasis on possible therapeutic applications.

A systematic literature search was performed in the MEDLINE/PubMed database for articles published between January 1990 and September 2015. The objective was to trace all literature containing original data on new findings and potential treatment strategies in the field of salivary gland SCs.

The literature search was performed in October 2015 using combinations of Mesh terms of “xerostomia”, “radiotherapy”, “salivary gland,” and “stem cell”. Furthermore, extensive cross‐referencing of the selected articles was performed. Case reports were eliminated from this review. Furthermore, the search was limited to English language. Gender and age were not limited.

## Prevention and treatment of radiation‐induced xerostomia

### Stem cell transplantation and bone marrow‐derived cell mobilization

#### Stem cell transplantation

Before transplantation of SCs is possible, surgical removal of salivary gland tissue before the start of the oncologic treatment is needed. Later on, SCs need to be collected. The ultimate goal of SC transplantation is regeneration of the function of the salivary gland by differentiation of these transplanted SCs into functional salivary gland cells 19, 20, 21, 22, 23, 24, 25.

Since 2004, several studies were performed in which submandibular and parotid gland SCs were transplanted in animal models after RT 19, 20, 21, 22, 23, 24, 25. The best marker, however, to select SCs for transplantation, still remains unclear. The most studied marker is *c‐Kit*. 15, 20, 21, 22, 25, 26 Transplantation of c‐Kit‐positive cells in mice submandibular glands can restore function and morphology 25, 26. Interestingly, these c‐Kit‐positive cells are also found in human salivary glands 26. Whether these c‐Kit‐positive cells in humans have the same regenerative potential needs to be investigated.

On the other hand, it is suggested that *CD 49f, CD 29, CD 24, and CD 133* might be used as markers for salivary gland SCs in mice 15, 27, 28, 29, 30. The finding of these other markers is important because some of the cells of the c‐Kit‐negative population may also have regenerative potential, indicating that c‐Kit is inadequate as a marker and that a combination of markers is needed, especially when a low amount of donor material is available 25.

Side population cells have been identified in various organs as putative SCs; their precise function, however, remains unclear. In mice salivary glands, side population cells did not seem to have SC activity 27. However, a side population cell‐derived protein—*clusterin*—was identified as a factor to recover from hypofunction of the salivary glands. It is believed that clusterin scavenges reactive oxygen species after irradiation 27. This means that this protein might have an additional benefit when stem cell transplantation is performed.

#### Bone marrow‐derived cell mobilization

Mesenchymal SCs from the bone marrow can be mobilized 31, 32. After migration, they secrete growth and survival factors that enhance the regeneration of tissue by stimulating the proliferation and differentiation of remaining salivary gland SCs and by decreasing inflammation and modifying the immune response 31. Importantly, this effect can be mimicked by adding keratinocyte growth factor, prophylactic pilocarpine (a nonselective muscarinic receptor agonist) or hyperbaric oxygen 23, 33, 34.

Selection of patients for this treatment will be very important. When the damage to the salivary gland is expected to be low, this technique using bone marrow‐derived cells could be enough to limit xerostomia after RT. When the damage is expected to be high, bone marrow‐derived cell mobilization could be insufficient, so SC transplantation may be necessary 31.

### Molecular regulation of salivary gland stem cells

Until recent, little was known about the molecular regulators of SCs in human salivary glands. Nowadays, there is growing evidence that the Wnt/*β*‐catenin signaling pathway is essential for maintenance and activation of different types of SCs, including these of the salivary glands 35, 36, 37. Regeneration of SCs is impaired by Wnt inhibition and enhanced by Wnt activation in epithelial organs such as the liver, airways, and intestines 38, 39. The central mediator of Wnt signaling is *β* catenin, which acts as an activator of gene transcription.

There is increasing data that activation of this pathway may also have a radioprotective effect and might decrease xerostomia numbers in irradiated patients 36, 40. Forced activation of the Wnt/*β*‐catenin pathway in mice during RT prevents both acute and chronic hyposalivation through inhibition of apoptosis and preservation of functional salivary stem/progenitor cells 36.

Wnt activation is also known to promote angiogenesis and innervation during development. In this regard, it is speculated that Wnt activation can help in the regeneration of the damage to blood supply and innervation after irradiation. This can play an additional role in the prevention of hyposalivation after RT 41.

These findings are promising; however, further research is mandatory to confirm the results and to investigate whether activation of this pathway is sufficient to prevent xerostomia in humans after RT.

Regarding the introduction of this technique, a major concern that needs further clarification is that overexpression of the Wnt/*β*‐catenin pathway has been linked to carcinogenesis 42.

Furthermore, there is emerging evidence that transient activation of the Hedgehog pathway and modulation of the GDNF pathway might play a role in preserving salivary gland SCs after RT 30, 43, 44, 45. An overview of all the cross talking intercellular signaling pathways involved in the development and regeneration of salivary gland SCs is beyond the scope of this study but can be found in a recent review by Liu et al. 43.

### Bath and shower principle in RT

Intensity‐modulated RT (IMRT) is the modality of choice to reduce xerostomia numbers following RT for HNC 12. Minimization of the mean dose to the parotid glands is the preferred technique to achieve parotid gland function sparing 13, 17. In spite of technical improvements, xerostomia after IMRT remains a serious problem 12.

A first possible explanation for this higher‐than‐expected incidence of xerostomia is the hypothesis that radiation of the parotid gland has a “bath and shower effect”. Photon‐based IMRT gives an overall low dose to the entire parotid gland 46. There is evidence that the tolerance to a high dose of RT to a small subvolume (“shower”) is strongly reduced by giving a subtolerance dose to the surrounding volume (“bath”) 46. Van Luijk et al. demonstrated this in the parotid gland in a rat model 46. The parotid glands were irradiated up to a dose of 10 Gy, which did not result in late loss of function. Addition of a bath of 1–10 Gy to the caudal 50% of the glands resulted in enhanced function loss. This indicates that avoiding an overall low dose to the parotid gland in rats could lead to less parotid gland hypofunction and subsequently to less xerostomia. Recovery after RT appears to be dependent on the number of remaining SCs after treatment 25. Currently, we have no good idea about the radiosensitivity of parotid gland SCs; however, the results above are suggesting that these SCs are very radiosensitive. High‐precision RT, avoiding low doses to the entire parotid gland, can possibly counteract this problem. Proton RT, for example, has a steeper dose gradient, and could therefore avoid a low dose to the surrounding tissues more easily.

### Sparing of SC‐rich regions in the parotid gland during RT

Sparing of SC‐rich regions within the parotid gland could help in preventing xerostomia following RT 47. The specific location of these SCs and therefore the region to spare has been the subject of debate. Several research groups found that (c‐Kit positive) stem/progenitor cells in the salivary gland are located in the larger excretory ducts 47, 48. Van Luijk et al. investigated the relationship between the localization of parotid SCs and late parotid gland dysfunction after irradiation in a rat model 46, 47. They found a correlation in the rat model where high‐precision irradiation to the center of the parotid gland resulted in excessive reduction of saliva production, indicating that this zone contains a large population of SCs. The dose to specific volumes of the parotid gland was also correlated by this research group to saliva production 1 year after RT. This research group showed in a cohort of patients with HNC that the RT dose to the region of the salivary gland containing stem/progenitor cells predicted the function of the salivary glands 1 year after RT 47. Miah et al. found that sparing the superficial lobe of both parotid glands may offer a higher incidence of recovery of salivary function compared to whole contralateral parotid gland sparing alone in oropharyngeal cancers 48. Buettner et al. found similar results 49. In our department, we conducted a study in which we included 28 patients who were treated with chemoradiotherapy (CRT) for HNC. We looked at the mean radiation dose to the superficial and deep ipsilateral parotid lobe and correlated this with patient‐ and physician‐scored xerostomia. In this small patient group, we observed a significant correlation between physician‐scored xerostomia ≥2 and the mean dose to the ipsilateral superficial parotid lobe. Sparing of the superficial ipsilateral parotid lobe, while sparing the whole contralateral parotid gland, could mean a step forward toward decreasing xerostomia after RT for HNC (unpublished data). We will investigate this hypothesis further in a larger patient cohort.

### Sparing of the submandibular gland and oral cavity during RT

Dijkema et al. stated that sparing of the contralateral submandibular gland, in addition to parotid gland sparing, might result in improved patient‐reported xerostomia 50. Little et al. found that mean doses to the parotid glands, submandibular glands, and oral cavity are significant predictors of both patient‐reported and observer‐rated xerostomia after chemo‐IMRT, with oral cavity doses remaining significant after adjusting for the parotid gland and submandibular gland doses 51. Jellema et al. found that the mean dose to the parotid and submandibular glands could influence the risk of xerostomia at 6 months; no significant differences were found when xerostomia was correlated with the oral cavity dose 9. These results, however, support efforts to spare all the salivary glands as much as possible 51. Mean RT doses to the submandibular gland exceeding 39 Gy cause permanent ablation of both stimulated and unstimulated flow 52. These findings are promising. It might be interesting to combine research on sparing of the submandibular gland and oral cavity with sparing of SC‐rich regions in the parotid gland during RT.

### Adaptive RT

One of the unique aspects of RT to the head and neck region is that noticeable changes in the anatomy occur during treatment. These changes include shrinkage of the tumor, but also of the surrounding organs at risk (such as the parotid and submandibular glands) 53. Furthermore, a medial shift of the parotid glands is often described during RT 54. A small study of our research group including five patients showed a median relative volume loss of the parotid glands of 41.5% during RT (range 20.0–48.4%). The parotid glands generally shifted medially due to tumor shrinkage and weight loss. Therefore, the plan created on the initial planning computed tomography may no longer be optimal for the changed anatomy during treatment. Moreover, the actual RT dose delivered to the patients may be significantly different from what was planned. Computed tomographies taken during the course of RT could be used to evaluate at what point in time the target volume, organs at risk and dose distribution have changed to such an extent that replanning is necessary 53. This adaptive approach could result in less irradiation of the organs at risk and thus less irradiation of the parotid SCs.

We recently closed a multicenter randomized adaptive radiotherapy trial including 100 patients with HNC. All patients received a computed tomography at 2 and 4 weeks after start of treatment, replanning was performed at these time‐points. Further follow‐up of these patients will give us more insights into whether this adaptive RT technique results in less xerostomia.

## Conclusions

To date, none of the described techniques concerning SCs to treat xerostomia are ready for clinical implementation, although most of them seem very promising in animal models.

It will become clear in future trials which technique or combination of techniques will be the most beneficial, cost‐effective, and easy to implement. The emphasis, nevertheless, has to be on avoiding xerostomia as much as possible. If both the shower and bath assumption, the role of the contralateral submandibular gland and the importance of SC sparing RT are confirmed in future trials, high‐specific proton RT could be a way to counteract these problems at the same time.

## Conflict of interest

None declared.
